# Protective Effects of Isoliquiritigenin and Licochalcone B on the Immunotoxicity of BDE-47: Antioxidant Effects Based on the Activation of the Nrf2 Pathway and Inhibition of the NF-κB Pathway

**DOI:** 10.3390/antiox13040445

**Published:** 2024-04-10

**Authors:** Minghui Dong, Ziying Yang, Qian Gao, Qingyuan Deng, Le Li, Hongmei Chen

**Affiliations:** 1 Key Laboratory of Xinjiang Phytomedicine Resources and Utilization, Ministry of Education, School of Pharmacy, Shihezi University, Shihezi 832002, China; 20212015028@stu.shzu.edu.cn (M.D.); yangziying@stu.shzu.edu.cn (Z.Y.); 20222515002@stu.shzu.edu.cn (Q.G.); dengqingyuan@stu.shzu.edu.cn (Q.D.); leele@shzu.edu.cn (L.L.); 2 Pharmacology Department, School of Pharmacy, Shihezi University, Shihezi 832002, China

**Keywords:** BDE-47, ISL, LCB, macrophage, protective effect, immune function

## Abstract

2,2′,4,4′-Tetrabrominated biphenyl ether (BDE-47) is a polybrominated diphenyl ether (PBDE) homologue that is ubiquitous in biological samples and highly toxic to humans and other organisms. Prior research has confirmed that BDE-47 can induce oxidative damage in RAW264.7 cells, resulting in apoptosis and impaired immune function. The current study mainly focused on how Isoliquiritigenin (ISL) and Licochalcone B (LCB) might protect against BDE-47’s immunotoxic effects on RAW264.7 cells. The results show that ISL and LCB could increase phagocytosis, increase the production of *MHC-II*, and decrease the production of inflammatory factors (TNF-α, IL-6, and IL-1β) and co-stimulatory factors (*CD40*, *CD80*, and *CD86*), alleviating the immune function impairment caused by BDE-47. Secondly, both ISL and LCB could reduce the expressions of the proteins Bax and Caspase-3, promote the expression of the protein Bcl-2, and reduce the apoptotic rate, alleviating the apoptosis initiated by BDE-47. Additionally, ISL and LCB could increase the levels of antioxidant substances (SOD, CAT, and GSH) and decrease the production of reactive oxygen species (ROS), thereby counteracting the oxidative stress induced by BDE-47. Ultimately, ISL and LCB suppress the NF-κB pathway by down-regulating IKBKB and up-regulating IκB-Alpha in addition to activating the Nrf2 pathway and promoting the production of HO-1 and NQO1. To summarize, BDE-47 causes oxidative damage that can be mitigated by ISL and LCB through the activation of the Nrf2 pathway and inhibition of the NF-κB pathway, which in turn prevents immune function impairment and apoptosis. These findings enrich the current understanding of the toxicological molecular mechanism of BDE-47 and the detoxification mechanism of licorice.

## 1. Introduction

2,2′,4,4′-Tetrabrominated biphenyl ether (BDE-47) is one of the polybrominated diphenyl ether homologues, which are widely used as fire retardants [1,2,3]. Studies have shown that BDE-47 exerts immunotoxicity, with negative effects on the immune system, by aggravating inflammation [4], suppressing immune function [5,6], and inducing immune cell apoptosis [4]. BDE-47 affects the production of immune cytokines in children and damages the complement system of the human innate immune system [5,6]. Similar phenomena also occur in marine organisms; for example, BDE-47 interferes with the innate immune system of Mytilus edulis and bivalve organisms, which adversely affects their survival [7,8]. Our previous research also confirmed that BDE-47 interferes with the immune system of mice, causing increases in immune cells and platelets, as well as decreases in the spleen index and thymus index [9]. In vitro studies have confirmed that BDE-47 induces immunotoxicity in RAW264.7 cells through the mitochondrial pathway, inducing apoptosis, decreasing phagocytotic function, increasing the levels of cellular inflammatory factors, and increasing the expressions of co-stimulatory factors [4]. All of the above studies have indicated that BDE-47 can impact the immune systems of organisms to varying degrees. Therefore, it is crucial to alleviate BDE-47-induced immunotoxicity. Further studies on the immunotoxic mechanisms of BDE-47 will help researchers to develop more targeted protective measures.

Studies have confirmed that BDE-47 affects the immune system by increasing inflammatory responses, regulating immune cell function, and regulating oxidative stress [4,5,6]. BDE-47 interferes with the activation of basophils by inhibiting proinflammatory cytokines in macrophages [10]. BDE-47 causes hemocyte death in Mytilus edulis by destabilizing the lysosomal membrane and reducing phagocytic ability [8]. In Ruditapes philippinarum, it reduces the viability of hemocytes, the granulocyte ratio, and phagocytotic activity [11]. Further studies showed that the immune impairment caused by BDE-47 is related to reactive oxygen species (ROS) imbalance as well as the NF-κB and Nrf2 pathways [8,11,12,13,14]. BDE-47 negatively regulates the NF-κB pathway, thereby inhibiting the immune responses of lymphocytes to LPS, thus disturbing the immune balance and increasing the body’s susceptibility to infectious diseases [15]. Furthermore, our previous studies confirmed that the immunotoxic effects of BDE-47 on RAW264.7 mouse macrophages are related to ROS imbalance, and Nrf2 was found to be a molecular target that could be used to reduce the immunotoxicity of BDE-47 [4,16]. Moreover, studies have reported that tert-butyl hydroquinone and sulforaphane also up-regulate antioxidant genes by activating the Nrf2/ARE signaling pathway, alleviating the oxidative stress and inflammation caused by BDE-47 in HTR-8/SVneo cells [16]. Based on previous studies, we boldly speculated that alleviating oxidative stress and inflammatory responses will reduce the immunotoxicity of BDE-47.

As a traditional Chinese medicine, licorice can alleviate the toxicity of many drugs. It has anti-inflammatory, detoxification, antioxidant, immune regulatory, and other functions [17,18]. Studies have shown that chalcones in licorice activate the Nrf2 signaling pathway and regulate the expressions of downstream antioxidant enzymes, having an antioxidant effect [19]. Chalcones have the potential to aid in the treatment of diseases mediated by oxidative stress and inflammation [19]. Isoliquiritigenin (ISL) and Licochalone B (LCB) are natural chalcone compounds that have anti-inflammatory and antioxidant effects. ISL suppresses inflammation by inhibiting the NF-κB, NLRP3, and MAPK pathways and activating the Nrf2 pathway [20]. ISL ameliorates BDE-47-induced developmental abnormalities in zebrafish [21]. It also alleviates the liver cell damage induced by tert-butyl hydrogen peroxide and liver toxicity caused by cadmium by activating the Nrf2 signaling pathway, through extracellular signal-regulated kinase [22]. LCB has alleviated neuronal injury in an MCAO rat stroke model through its antioxidant effect and by stimulating the Nrf2 pathway [23].

There are currently few studies on drugs for alleviating the immunotoxicity of BDE-47. To fill this gap, an in vitro model of RAW264.7 cells exposed to BDE-47 was established to assess how well ISL and LCB protect against BDE-47-induced immunotoxicity in terms of apoptosis, inflammation, and redox processes. Moreover, the molecular mechanisms underlying ISL’s and LCB’s abilities to protect RAW264.7 cells against toxicity were further elucidated.

## 2. Materials and Methods

### 2.1. Materials

BDE-47 (≥99.9%) was obtained from CSNpharm (Chicago, IL, USA). ISL (≥98%, HPLC) and LCB (≥98%, HPLC) were obtained from Yuanye (Shanghai, China). DCFH-DA and DMSO were obtained from Solarbio (Beijing, China). An apoptosis kit was obtained from Multi science (Hangzhou, China). A DAPI dye and neutral red staining solution were obtained from Beyotime Biotechnology (Shanghai, China). ELISA kits were obtained from Mlbio (Shanghai, China) (TNF-α Cat. No. ml002095; IL-6 Cat. No. ml063159; IL-1β Cat. No. ml301814). CAT, SOD, and GSH kits were obtained from Solarbio (Beijing, China). β-Actin, Nrf2, Keap1, HO-1, NQO1, IKBKB, IκB-Alpha, Bax, Bcl-2 antibodies, and the ECL luminescent reagent were obtained from Proteintech (Wuhan, China) (β-Actin Cat. No. 66009-1-lg; Nrf2 Cat. No. 16396-1-AP; Keap1 Cat. No. 60027-1-lg; HO-1 Cat. No. 66743-1-lg; NQO1 Cat. No. 67240-1-lg; IKBKB Cat. No. 15649-1-AP; IκB-Alpha Cat. No. 10268-1-AP; Bax Cat. No. 50599-2-lg; Bcl-2 Cat. No. 26593-1-AP). Caspase-3 antibody was obtained from BBI (Shanghai, China) (Cat. No. D16009). NF-κB and p-NF-κB antibodies were obtained from Abcam (Cambridge, UK) (NF-κB Cat. No. ab76302; p-NF-κB Cat. No. ab32536). P-Nrf2 antibody was obtained from Abclonal (Wuhan, China) (Cat. No. AP1133). Real-time fluorescent quantitative reagents were obtained from TransGen Biotech (Beijing, China).

### 2.2. Cell Culture and Experimental Design

Cells of the murine macrophage cell line RAW264.7 were obtained from the Shanghai Institute of Cell Biology (Shanghai, China). The cells were incubated in DMEM containing 10% FBS and 1% penicillin–streptomycin antibiotics. Then, they were placed in an incubator at 37 °C and 5% CO_2_.

A 100 μL RAW264.7 cell suspension with a concentration of 1.2 × 10^5^ mL^−1^ was added to a 96-well plate. A total of 1 mL of RAW264.7 cell suspension at 10^6^ mL^−1^ was added into a 6-well plate. There were eight groups in the experiment as follows: (1) DMEM-treated control group (Control); (2) BDE-47 (40 μM)-treated group (BDE-47); (3) low-dose (5 μM) ISL-treated cells exposed to BDE-47 (BDE-47 + 5 μM ISL); (4) middle-dose (10 μM) ISL-treated cells exposed to BDE-47 (BDE-47 + 10 μM ISL); (5) high-dose (15 μM) ISL-treated cells exposed to BDE-47 (BDE-47 + 15 μM ISL); (6) low-dose (0.25 μM) LCB-treated cells exposed to BDE-47 (BDE-47 + 0.25 μM LCB); (7) middle-dose (0.5 μM) LCB-treated cells exposed to BDE-47 (BDE-47 + 0.5 μM LCB); and (8) high-dose (0.75 μM) LCB-treated cells exposed to BDE-47 (BDE-47 + 0.75 μM LCB).

### 2.3. Cell Viability Assay

The cells were treated with various concentrations of BDE-47 (0–100 μM), ISL (0–40 μM), or LCB (0–40 μM) for 24 h. After adding 10 μL of 5 mg/mL MTT solution to each well, the culture plate was incubated for 4 h at 37 °C. Then, 100 μL of DMSO was added to dissolve the formazan. Finally, the OD value of the solution at 450 nm was detected using an enzyme marker.

### 2.4. Phagocytic Capacity of Cells 

Neutral erythrophagocytosis is an experimental method commonly used to study the function of cell phagocytosis [24]. This method quantifies phagocytic activity according to the intake of a neutral red substance via phagocytosis and color changes in the cells; cells showing strong phagocytosis take in more of the substance, making the interior of the cell more red.

After the cells were exposed to BDE-47 and/or ISL/LCB, 1 mL of neutral red staining solution was added for a 10 min incubation. The cells were washed with PBS until there was no visible red color in the wells. Then, the staining of cells was observed with a positive microscope.

### 2.5. Enzyme-Linked Immunosorbent (ELISA) Assay 

An enzyme-linked immunosorbent assay was employed to quantify the contents of TNF-α, IL-6, and IL-1β.

### 2.6. Observation of Nuclear Morphology

According to the manufacturer’s method, 1 mL of 10 μg/mL DAPI solution was added to each well, and the plate was incubated in a constant temperature incubator at 37 °C for 20 min. Then, the cells were collected, and the staining of the nuclei was observed with a positive fluorescence microscope.

### 2.7. Apoptotic Rate Detection

The cells were separated and suspended in 500 μL of binding buffer containing Annexin V-FITC/PI according to the manufacturer’s method. After incubation at 37 °C for 15 min, the apoptotic rates were determined via flow cytometry, using FlowJo (v10.6.2) to analyze the data. The apoptotic rate was expressed as the sum of the percentages in the Q2 and Q3 quadrants.

### 2.8. Western Blotting Assay 

The total protein of RAW264.7 cells was extracted through lysis with a RIPA-to-PMSF ratio of 100:1, and the protein concentration was determined with a BCA protein assay kit. The protein was separated using 12% SDS-PAGE. PVDF membranes were used during the membrane transfer process. Under RT conditions, 5% (*w*/*v*) skimmed milk was dissolved in a triple buffer containing 0.1% tween-20 (TBST), left to stand for at least 1 h, and then incubated overnight with an appropriate primary antibody at 4 °C. On the second day, horseradish peroxidase-conjugated antibodies (1:10,000) were incubated with the membrane under RT conditions for 1 h. ECL luminescent droplets were placed on the PVDF membrane and developed using a chemiluminescence instrument after waiting for a few minutes. The experimental data were analyzed using ImageJ software v 1.53. The expression of the target protein was normalized using β-actin.

### 2.9. Examination of ROS Levels

After the 24 h treatment of cells in different groups, a DCFH-DA fluorescent probe was added to each well and left to incubate with them at 37 °C for 30 min. The obtained samples were analyzed through BD flow cytometry. The special fluorescence of the cells was observed with a Carl Zeiss positive fluorescence microscope (Oberkochen, Germany).

### 2.10. Analysis of Antioxidant Substance Activity

The standard activity levels of catalase (CAT), superoxide dismutase (SOD), and antioxidant glutathione (GSH) in the cell samples were detected using a series of commercial test kits under the guidance of the product manual. 

### 2.11. Quantitative Real-Time Polymerase Chain Reaction (QRT-PCR) Analysis

Total RNA was extracted from RAW264.7 cells using RNA extraction reagents according to the manufacturer’s instructions. Then, the RNA was reverse transcribed into cDNA using a Reverse Transcription kit from TransGen Biotech for the total RNA. The samples were subjected to polymerase chain reactions with SYBR premix ex Taq with a fluorescence quantitative PCR instrument. The sequences of primers used in the real-time PCR are listed in Table 1. The relative quantification of mRNA was performed using the 2^−ΔΔCt^ method, and GAPDH was used for normalization.

### 2.12. Statistical Analysis

In this study, each experiment was repeated three times independently with similar results, and representative data are shown. The experimental data are presented as the mean ± standard error (mean ± SD) and were statistically analyzed using a two-tailed Student’s *t*-test for unpaired data. *p*-values less than 0.05 were considered statistically significant. *p*-values for interactions represent significant differences between two groups of suppression curves in a panel, which were calculated through two-way ANOVA using GraphPad Prism 8. * represents a significant difference compared to the control group, and ^#^ represents a significant difference compared to the BDE-47 group.

## 3. Results

### 3.1. ISL and LCB Alleviate BDE-47’s Cytotoxic Effects on RAW264.7

**Figure 1:**
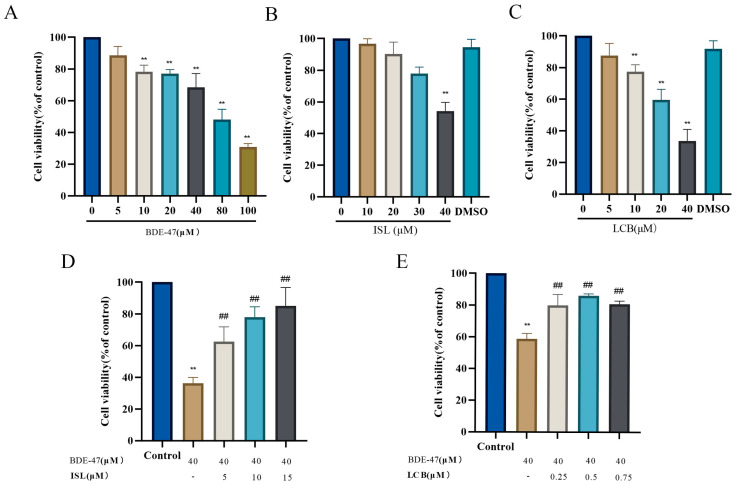
Effects of 2,2′,4,4′-tetrabrominated biphenyl ether (BDE-47), Isoliquiritigenin (ISL), Licochalcone B (LCB) alone, and BDE-47 combined with ISL and LCB on RAW264.7 cell viability. (**A**–**C**) Effects of BDE-47 (0–100 μM), ISL (0–40 μM), and LCB (0–40 μM) on cell viability. (**D**) Effects of different concentrations of ISL combined with BDE-47 on cell viability. (**E**) Effects of different concentrations of LCB combined with BDE-47 on cell viability. There was a significant difference compared to the control group (** *p* < 0.01). There was a significant difference compared to the BDE-47 group (^##^ *p* < 0.01).

With an increase in BDE-47 concentration, the viability of RAW264.7 cells decreased in a dose-dependent manner. When the cells were treated with BDE-47 at a concentration higher than 10 μM for 24 h, the proliferation of the cells was significantly decreased (*p* < 0.01) (Figure 1A). Treating the cells with 10, 20, and 30 μM ISL and 5 μM LCB for 24 h had no significant effect on cell viability (Figure 1B,C). Therefore, 40 μM BDE-47; 5, 10, and 15 μM ISL; and 0.25, 0.5, and 0.75 μM LCB were used for the subsequent experiments.

As shown in Figure 1D, co-treatment with 5, 10, and 15 μM ISL and BDE-47 resulted in significantly higher viability levels than treatment with BDE-47 (40 μM) alone (*p* < 0.01). Similar results are shown in Figure 1E, wherein co-treatment with 0.25, 0.5, and 0.75 μM LCB and BDE-47 also resulted in significantly higher viability levels than BDE-47 (40 μM) treatment alone (*p* < 0.01). The results show that ISL and LCB could alleviate BDE-47’s cytotoxic effects on RAW264.7.

### 3.2. ISL and LCB Ameliorate BDE-47-Induced Immune Impairment

**Figure 2:**
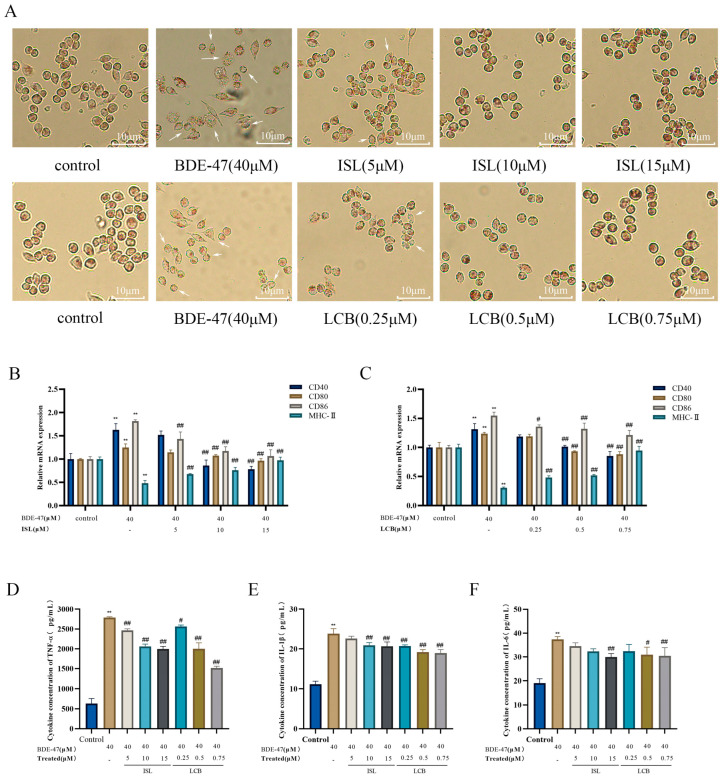
The effect of ISL and LCB combined with BDE-47 on the immune function of RAW264.7 cells. (**A**) Neutral red staining under an upright microscope (200×). The arrows point to cells with impaired phagocytic function. The effects of (**B**) ISL and (**C**) LCB combined with BDE-47 on the expression levels of cell antigen presentation genes. (**D**–**F**) The effects of the combination of ISL and LCB with BDE-47 on the secretion of inflammatory factors in cells. There was a significant difference compared to the control group (** *p* < 0.01). There was a significant difference compared to the BDE-47 group (^#^ *p* < 0.05, ^##^ *p* < 0.01).

Neutral red staining experiments were performed to observe the changes in the phagocytic ability of cells in different groups at the same staining time. At the same staining time, the phagocytic abilities of RAW264.7 cells in the control, BDE-47, and ISL/LCB combined with BDE-47 groups were different. The red color of the cells in the BDE-47 group was lighter than that in the control group, and unstained cells appeared. This indicated that the BDE-47 group had weaker phagocytosis and several damaged cells. Compared with that in the BDE-47 group, the red color gradually deepened in the cells treated with the low, medium, and high concentrations of ISL and LCB in combination with BDE-47. This indicates that the cells in the groups treated with different concentrations of ISL and LCB combined with BDE-47 showed improved phagocytosis. The phagocytosis of cells in the high-concentration groups (BDE-47 + 15 μM ISL; BDE-47 + 0.75 μM LCB) was significantly improved (Figure 2A).

Compared with that in the control group, the expression of *MHC-II* was dramatically reduced (*p* < 0.01) through treatment with BDE-47, whereas the expressions of costimulatory factors *CD40*, *CD80*, and *CD86* were significantly increased (*p* < 0.01). The BDE-47 + 5 μM ISL, BDE-47 + 10 μM ISL, and BDE-47 + 15 μM ISL groups all showed reduced elevations in *CD40*, *CD80*, and *CD86* and increased *MHC-II* in comparison to the BDE-47 group. Similarly, the BDE-47 + 0.25 μM LCB, BDE-47 + 0.5 μM LCB, and BDE-47 + 0.75 μM LCB groups showed reduced elevations in *CD40*, *CD80*, and *CD86* and increased *MHC-II*. The group with high concentrations (BDE-47 + 15 μM ISL; BDE-47 + 0.75 μM LCB) showed the greatest improvement (*p* < 0.01) (Figure 2B,C). These results indicate that ISL and LCB can alleviate the BDE-47-induced disruption of immune markers.

BDE-47 significantly promoted the release of inflammatory factors (TNF-α, IL-6, and IL-1β) (Figure 2D–F). Compared with those in the control group, their releases were elevated by 2159.1 pg/mL, 18.21 pg/mL, and 12.66 pg/mL, respectively. Meanwhile, the acting concentrations of both ISL and LCB alleviated the BDE-47-induced releases of the three factors to varying degrees. Compared with the levels in the BDE-47 group, the three factors were reduced by 327 pg/mL, 2.85 pg/mL, and 1.22 pg/mL in the low-concentration group (BDE-47 + 5 μM ISL), and 226 pg/mL, 4.93 pg/mL, and 3.11 pg/mL in the BDE-47 + 0.25 μM LCB group. The middle-concentration group (BDE-47 + 10 μM ISL) showed reductions in the three factors of 729 pg/mL, 5.02 pg/mL, and 2.94 pg/mL, respectively, and the BDE-47 + 0.5 μM LCB group showed reductions of 789 pg/mL, 6.4 pg/mL, and 4.63 pg/mL, respectively. The high-concentration group resulted in the greatest relief, with the BDE-47 + 15 μM ISL group showing decreases in the three proinflammatory factors of 794 pg/mL, 3.16 pg/mL, and 7.38 pg/mL. Meanwhile, the BDE-47 + 0.75 μM LCB group showed decreases in these factors of 1273 pg/mL, 4.85 pg/mL, and 6.93 pg/mL. The results show that ISL and LCB could alleviate the oversecretion of inflammatory cytokines in BDE-47-infected cells.

### 3.3. ISL and LCB Alleviate BDE-47-Induced Apoptosis 

**Figure 3:**
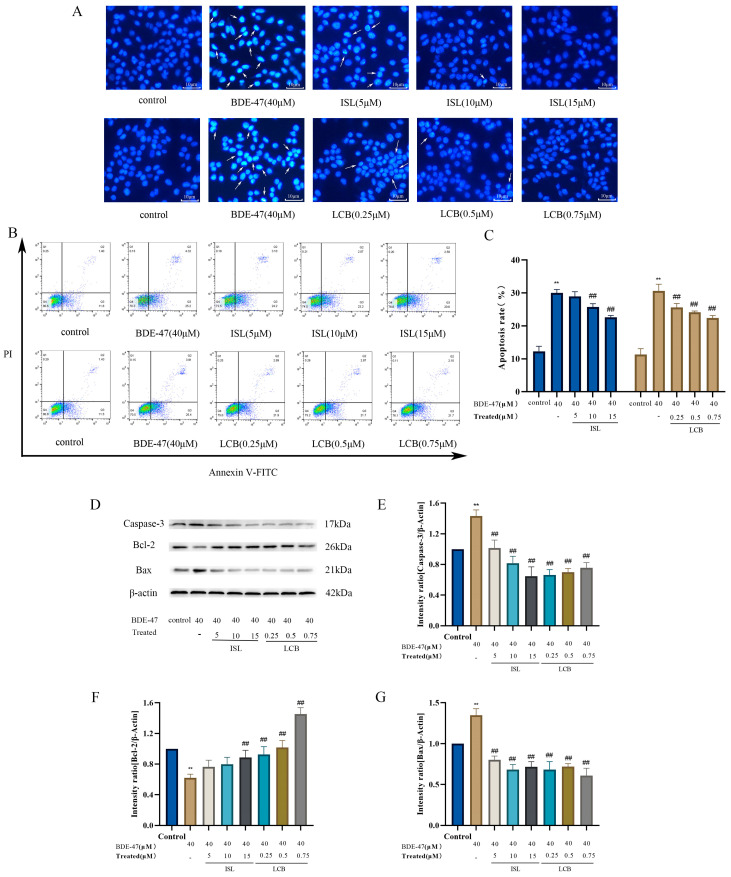
The effects of ISL and LCB combined with BDE-47 on the apoptosis of RAW264.7 cells. (**A**) DAPI staining under an upright fluorescence microscope (200×) camera. The arrows point to cells with obvious apoptosis. (**B**) The representative cell apoptosis chart and (**C**) quantitative bar chart obtained from flow cytometry analysis. (**D**) The band patterns of the apoptotic proteins Caspase-3, Bax, and Bcl-2. (**E**–**G**) The quantitative statistics of the proteins. There was a significant difference compared to the control group (** *p* < 0.01). There was a significant difference compared to the BDE-47 group (^##^ *p* < 0.01).

DAPI staining showed that the cells in the control group exhibited uniform blue fluorescence, while the BDE-47 group exhibited bright blue fluorescence accompanied by nuclear shrinkage and fragmentation. A small amount of bright blue fluorescence was observed in the group treated with a low concentration of ISL and LCB combined with BDE-47 (BDE-47 + 5 μM ISL; BDE-47 + 0.25 μM LCB), while the group treated with a high concentration of ISL and LCB combined with BDE-47 (BDE-47 + 15 μM ISL; BDE-47 + 0.75 μM LCB) exhibited uniform blue fluorescence. The high concentrations of ISL and LCB could alleviate BDE-47-induced RAW264.7 cell apoptosis (Figure 3A).

The results of the flow cytometry analysis show that BDE-47 significantly increased the apoptosis rate (*p* < 0.01), with the apoptosis rates increasing from 12.27% ± 1.27% to 30.03% ± 0.84% and from 11.32% ± 1.45% to 30.63% ± 1.65%. Compared with those in the BDE-47 group, the apoptotic rates in the groups treated with ISL and LCB at different concentrations in combination with BDE-47 showed a decreasing trend and were dose-dependent. The apoptotic rates when 10 and 15 μM ISL were used in combination with BDE-47 (BDE-47 + 10 μM ISL; BDE-47 + 15 μM ISL) were considerably (*p* < 0.01) lower than the rate in the BDE-47 group, showing decreases of 4.28% ± 0.03% and 7.43% ± 0.98%. The rates of apoptosis observed following treatment with LCB at concentrations of 0.25, 0.5, and 0.75 μM combined with BDE-47 (BDE-47 + 0.25 μM LCB; BDE-47 + 0.5 μM LCB; BDE-47 + 0.75 μM LCB) were significantly lower than those in the BDE-47 treatment group (*p* < 0.01) (Figure 3B,C). The apoptotic rate in the group treated with LCB at 0.75 μM combined with BDE-47 (BDE-47 + 0.75 μM LCB) was 8.22% ± 2.25% lower than that in the BDE-47 group.

After BDE-47 acted upon the cells, the expressions of the apoptosis promoter Caspase-3 and pro-apoptotic protein Bax significantly increased (by 43.26% and 34.78%, respectively) (*p* < 0.01). However, the expression of the anti-apoptotic protein Bcl-2 was significantly decreased (by 38.04%) (*p* < 0.01). In following the combination of ISL and LCB with BDE-47 at various doses, the expressions of Caspase-3 and Bax dropped, and the expression of Bcl-2 increased in comparison to those in the BDE-47 group. The high-concentration group showed a large effect. Compared with the BDE-47 group, the BDE-47 + 15 μM ISL and BDE-47 + 0.75 μM LCB groups showed decreases in Caspase-3 expression of 78.67% and 67.61%, respectively; decreases in Bax expression of 63.13% and 73.95%; and increases in Bcl-2 expression of 26.89% and 83.21%, respectively. The results show the same trend as that of the apoptosis rate, indicating that ISL and LCB could ameliorate the apoptosis of RAW264.7 cells induced by BDE-47 (Figure 3D–G).

### 3.4. ISL and LCB Alleviate BDE-47-Induced Oxidative Stress

**Figure 4:**
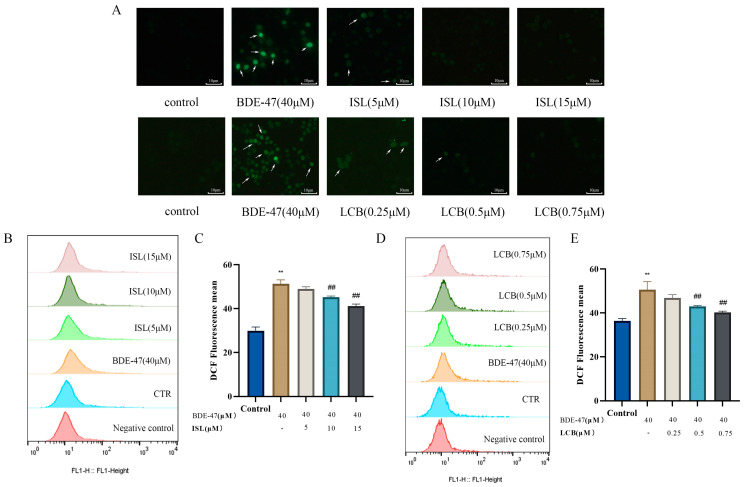
The effects of BDE-47 alone or in combination with ISL and LCB on the levels of ROS in RAW264.7 cells. (**A**) DCFH-DA staining under an upright fluorescence microscope (200×) camera. The arrows point to cells with an obvious fluorescence phenomenon. (**B**,**C**) Representative map and quantitative data obtained with flow cytometry analysis for ROS in RAW264.7 cells treated with ISL and (**D**,**E**) LCB. There was a significant difference compared to the control group (** *p* < 0.01). There was a significant difference compared to the BDE-47 group (^##^ *p* < 0.01).

After the BDE-47 treatment of RAW264.7 cells for 24 h, the BDE-47 group showed an increase in the number of green fluorescent cells and a significant accumulation of intracellular reactive oxygen species compared with the control group. The low-concentration groups (BDE-47 + 5 μM ISL and BDE-47 + 0.25 μM LCB-treated cells) showed a slight decrease in green fluorescent cells and a decrease in intracellular reactive oxygen species. After the cells in the medium- and high-concentration groups were treated (BDE-47 + 10 μM ISL, BDE-47 + 15 μM ISL, BDE-47 + 0.5 μM LCB, and BDE-47 + 0.75 μM LCB), the number of green fluorescent cells was significantly reduced and the accumulation of intracellular reactive oxygen species was significantly decreased (Figure 4A).

The flow cytometry results show that ROS levels were significantly higher in the BDE-47 group compared to the control group (*p* < 0.01), with the mean fluorescence values being elevated by 21.37% ± 2.90% and 14.30% ± 3.28%, respectively. The ROS levels showed a decreasing trend and a dose-dependent effect after the combination of two different concentrations of the drugs with BDE-47. The ROS levels were reduced in the low-concentration BDE-47 + 5 μM ISL and BDE-47 + 0.25 μM LCB groups, and the values were reduced by 2.27% ± 2.89% and 3.77% ± 2.11% compared to those in the BDE-47 group, which was not significant. The medium-concentration BDE-47 + 10 μM ISL and BDE-47 + 0.5 μM LCB groups had significantly decreased ROS levels (*p* < 0.01), with reductions of 6.00% ± 1.76% and 7.67% ± 2.66%, respectively. Remarkably, the high-concentration group showed the most significant decreases in ROS, with values that were 10.07% ± 1.83% and 10.43% ± 3.01% lower in the BDE-47 + 15 μM ISL and BDE-47 + 0.75 μM LCB groups (Figure 4B–E). To summarize, ISL and LCB were able to reduce the elevation in ROS induced by BDE-47.

**Figure 5:**
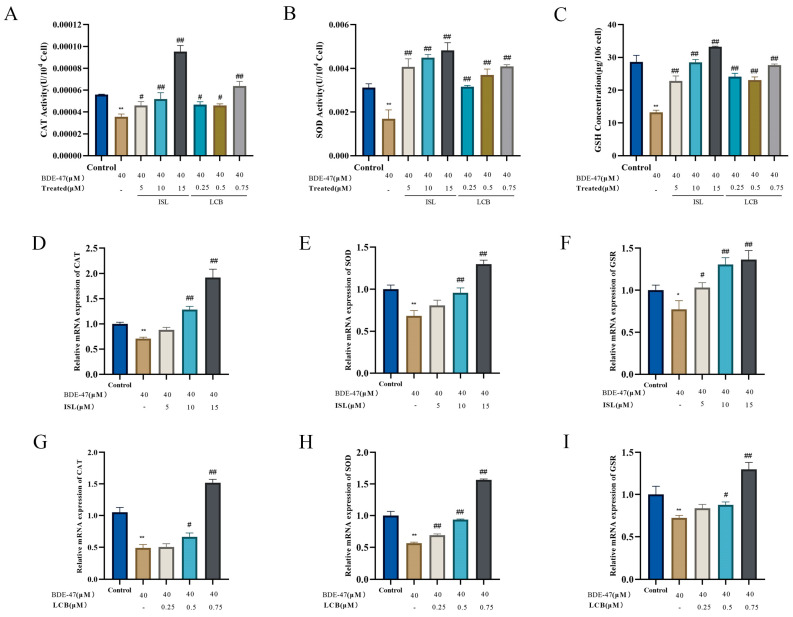
The effects of BDE-47 alone or in combination with ISL and LCB on the levels of antioxidant substances in RAW264.7 cells. (**A**–**C**) Activity levels of CAT, SOD, and GSH. (**D**–**F**) mRNA levels of *CAT*, *SOD*, and *GSR* in BDE-47 infected cells induced by ISL. (**G**–**I**) mRNA levels of *CAT*, *SOD*, and *GSR* in BDE-47 infected cells induced by LCB. There was a significant difference compared to the control group (* *p* < 0.05, ** *p* < 0.01). There was a significant difference compared to the BDE-47 group (^#^ *p* < 0.05, ^##^ *p* < 0.01).

Furthermore, the BDE-47 treatment of RAW264.7 cells significantly reduced the catalase activity, superoxide dismutase activity, and reduced glutathione content (*p* < 0.05) (Figure 5A–C). In contrast, when combined with BDE-47, ISL and LCB could differentially increase their activities. Compared with the BDE-47 group, the low-concentration groups (BDE-47 + 5 μM ISL and BDE-47 + 0.25 μM LCB) showed increases in catalase activity, superoxide dismutase activity, and glutathione content of (1.04 × 10^−5^ ± 3.45 × 10^−5^) U/10^4^ cells and (1.11 × 10^−5^ ± 4.28 × 10^−5^) U/10^4^ cells; (2.79 × 10^−3^ ± 2.04 × 10^−4^) U/10^4^ cells and (1.46 × 10^−3^ ± 2.94 × 10^−4^) U/10^4^ cells; and (9.57 ± 1.69) pg/10^6^ cells and (10.88 ± 1.39) pg/10^6^ cells, respectively. The medium-concentration group (BDE-47 + 10 μM ISL; BDE-47+ 0.5 μM LCB) showed further increases in catalase activity, superoxide dismutase activity, and glutathione content. The high-concentration group (BDE-47 + 15 μM ISL; BDE-47 + 0.75 μM LCB) showed significant increases in catalase activity, superoxide dismutase activity, and glutathione content (*p* < 0.01).

The intracellular mRNA expressions of -*CAT*, *SOD*, and *GSR* were detected using *RT-qPCR*. RAW264.7 cells treated with BDE-47 showed varying decreases in the intracellular *CAT*, *SOD*, and *GSR* mRNA expressions (*p* < 0.05) (Figure 5D–I). Compared with the BDE-47 group, the low-concentration BDE-47 + 5 μM ISL and BDE-47 + 0.25 μM LCB groups showed no significant effects on the mRNA expression of *CAT*. The BDE-47 + 5 μM ISL group showed no significant effect on the mRNA expression of *SOD*. The mRNA expression of *GSR* was not significantly affected in the BDE-47 + 0.25 μM LCB group. This indicates that the low concentration had little effect on the mRNAs for the three antioxidant substances. However, the medium- and high-concentration groups (BDE-47 + 10 μM ISL; BDE-47 + 0.5 μM LCB; BDE-47 + 15 μM ISL; BDE-47 + 0.75 μM LCB) showed significant increases in the mRNA expressions of the three antioxidant substances (*p* < 0.05). The results show that ISL and LCB could promote the secretion of antioxidant substances in BDE-47-infected cells. In summary, ISL and LCB alleviated the cellular oxidative stress induced by BDE-47.

### 3.5. ISL and LCB Activate the Nrf2 Pathway and Inhibit the NF-ĸB Pathway

**Figure 6:**
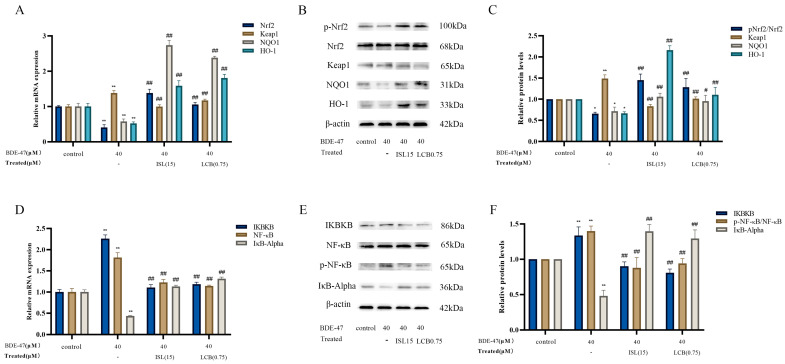
Effects of the combination of ISL and LCB with BDE-47 on the expressions of genes and proteins associated with the Nrf2 and NF-κB pathways. (**A**) Expressions of Nrf2-pathway-related genes. (**B**) Strip diagram of Nrf2-pathway-related proteins. (**C**) Quantitative statistics diagram for Nrf2-pathway-related proteins. (**D**) Expressions of NF-κB-pathway-related genes. (**E**) Strip diagram for NF-κB-pathway-related proteins. (**F**) Quantitative statistics diagram for NF-κB-pathway-related proteins. There was a significant difference compared to the control group (* *p* < 0.05, ** *p* < 0.01). There was a significant difference compared to the BDE-47 group (^#^ *p* < 0.05, ^##^ *p* < 0.01).

BDE-47 significantly decreased the mRNA levels of *Nrf2*, *HO-1*, and *NQO1* compared with those in the control group (*p* < 0.01), while it significantly increased the *Keap1* mRNA expression level (*p* < 0.01) (Figure 6A). Combining 15 μM ISL with the BDE-47 treatment and 0.75 μM LCB with the BDE-47 treatment (BDE-47 + 15 μM ISL; BDE-47 + 0.75 μM LCB) could revert the above mRNA levels to varying degrees. A WB assay of the intracellular expressions of five proteins was used to assess the effect of ISL and LCB on the Nrf2 pathway in BDE-47-stained RAW264.7 cells. We found that the BDE-47 stimulation of RAW264.7 cells resulted in significant down-regulations of the p-Nrf2/Nrf2, HO-1, and NQO1 protein expressions (by 34.2%, 33.3%, and 28.2%, respectively) (*p* < 0.05); the Keap1 protein was significantly up-regulated (by 49.2%) (*p* < 0.01). These improved when cells were treated with ISL and LCB. We found that the expression of p-Nrf2/Nrf2 in the BDE-47 + 15 μM ISL group was up-regulated by 79.24% (*p* < 0.01), and the expression of Keap1 decreased by 65.75% (*p* < 0.01). In addition, the expressions of HO-1 and NQO1 downstream of the Nrf2 pathway were up-regulated by 149.20% and 34.12% (*p* < 0.01). The BDE-47 + 0.75 μM LCB group also showed the same phenomenon as the BDE-47 + 15 μM ISL group. The p-Nrf2/Nrf2 expression in the BDE-47 + 0.75 μM LCB group was up-regulated by 62.85% (*p* < 0.01), and the expression of Keap1 was decreased by 47.97% (*p* < 0.01). Meanwhile, the expressions of HO-1 and NQO1 were increased by 43.49% and 23.50% (*p* < 0.05), respectively. The above results indicate that ISL and LCB could alleviate BDE-47-induced cellular injury by activating the Nrf2 pathway.

The gene expressions of *IKBKB* and *NF-κB* in the BDE-47 group were significantly elevated (*p* < 0.01), while the *IκB-Alpha* gene expression level was reduced in the BDE-47 group (*p* < 0.01) (Figure 6D). Combining 15 μM ISL with BDE-47 (BDE-47 + 15 μM ISL) and 0.75 μM LCB with BDE-47 (BDE-47 + 0.75 μM LCB) could improve the effects of the BDE-47 treatment on gene expression levels to varying degrees. To investigate the effects of ISL and LCB on the NF-κB pathway, we detected the proteins of IKBKB, IκB-Alpha, NF-κB, and p-NF-κB. As demonstrated in Figure 6E,F, the BDE-47 stimulation of RAW264.7 cells resulted in significant up-regulations of the IKBKB and p-NF-κB/ NF-κB protein expressions (by 33.6% and 39.9%, respectively) (*p* < 0.01), as well as a down-regulation of the IκB-Alpha protein expression (by 52%, *p* < 0.01). Notably, ISL and LCB significantly down-regulated the IKBKB and p-NF-κB/NF-κB protein expressions (*p* < 0.01), and significantly increased the IκB-Alpha protein expression (Figure 6C). Differently, combining 15 μM ISL with BDE-47 (BDE-47 + 15 μM ISL) down-regulated the IKBKB and p-NF-κB/NF-κB proteins by 40.74% and 52.09%, whereas combining 0.75 μM LCB with BDE-47 (BDE-47 + 0.75 μM LCB) reduced them by 52.75% and 45.88%. On the other hand, the IκB-Alpha protein expression was up-regulated by 91.68% in the group where 15 μM ISL was combined with BDE-47 (BDE-47 + 15 μM ISL) and by 81.30% in the group where 0.75 μM LCB was combined with BDE-47 (BDE-47 + 0.75 μM LCB). The *RT-qPCR* results are consistent with the protein detection results, indicating that ISL and LCB could relieve BDE-47-induced inflammatory responses by inhibiting the NF-κB pathway, thereby alleviating cell damage.

## 4. Discussion

Oxidative stress is not only the cause of many diseases, but also one of the factors that promote the development of diseases. Studies have shown that oxidative stress is closely related to cancer, high blood pressure, diabetes, skin damage, and other diseases [25,26,27,28]. Therefore, antioxidants also become an entry point for disease treatment. In our previous studies, we confirmed that oxidative stress induced by PBDE homologues such as BDE-47 leads to immunotoxicity in mice and RAW264.7 mouse macrophages, and Park confirmed that Nrf2 is a molecular target for combating the immunotoxicity of BDE-47 [4,16]. Therefore, the regulation of redox balance and activation of the Nrf2 pathway may mitigate the immunotoxicity caused by BDE-47. Various active components of glycyrrhiza can play a protective role by regulating redox balance [29,30,31]. In this study, normal RAW264.7 cells were selected as experimental subjects, and the protective effects of ISL and LCB on the immunotoxicity of BDE-47 and underlying mechanisms were explored by focusing on inflammation, apoptosis, and redox mechanisms based on immune cytotoxicity.

In this study, 5–100 μM BDE-47 was applied to RAW264.7 for 24 h, and the findings demonstrate a concentration-dependent decline in cell viability as the BDE-47 concentration increased [32,33,34]. However, after the different concentrations of ISL and LCB used in this study were applied, the cell viability was improved to varying degrees. Hence, we propose that ISL and LCB can effectively alleviate BDE-47-induced cytotoxicity in RAW264.7, which is worthy of further study.

Foreign poisons interact with the immune system after entering the body, resulting in a decrease in the number of immune cells, interfering with the normal function of immune cells, and damaging the immune system. We evaluated the effects of BDE-47 alone and ISL and LCB combined with BDE-47 on the immune function of RAW264.7 cells by examining the phagocytosis, antigen-presenting gene expression, and proinflammatory factor secretion of macrophages. Our research suggests that poisoning cells with 40 μM BDE-47 can decrease their ability to phagocytose neutrophils; increase the expressions of the cytokines *CD40*, *CD80*, and *CD86* at the mRNA level; and reduce the expression of *MHC-II*, thereby altering the antigen presentation ability of RAW264.7 cells. In addition, it can promote the expressions of inflammatory factors within cells. The combination of medium and high concentrations of ISL and LCB with BDE-47 can significantly improve the above phenomena, lessening the immune function impairment that BDE-47 causes. We have confirmed in previous studies that BDE-47 diminishes RAW264.7 cells’ ability to phagocytose and their expression of antigen-presenting molecules [4]. In addition, Tang Jie’s research discovered that exposure to BDE-47 can up-regulate IL-1β, IL-6, and TNF-α in cochlear hair cells (HEI-OC1) [35]. Moreover, when HKC cells were exposed to this organic pollutant, the increase in apoptosis was related to the NLRP3 inflammasome and the pathway of inflammatory cell death [36]. In other studies, in in vitro models of RAW264.7 cells infected with mycobacterium tuberculosis, ISL treatment significantly reduced the levels of IL-6 and IL-1β [37]. In in vitro models of RAW264.7 cells induced by LPS, ISL/LCB treatment significantly reduced the mRNA expressions of IL-6 and IL-1β [38,39].

After 24 h of exposure to BDE-47, the apoptosis rate of RAW264.7 cells was significantly increased, the expressions of the apoptosis promoter Caspase-3 and pro-apoptotic protein Bax were significantly increased, and the expression of the anti-apoptotic protein Bcl-2 was significantly decreased, indicating that BDE-47 could induce apoptosis in RAW264.7 cells. In other similar studies, BDE-47 has induced apoptosis in liver cells, kidney cells, and gonadal cells [40,41,42]. It also causes apoptosis in zebrafish embryos, Marine Rotifers, planarias, and Intertidal Crabs [43,44,45,46]. It is worth noting that ISL and LCB can alleviate the BDE-47-induced apoptosis of RAW264.7 cells to varying degrees. At the same time, the increases in Caspase-3 and Bax induced by BDE-47 were alleviated, and the expression of Bcl-2 was significantly increased. This detoxification effect was observed with the combination of ISL and LCB at medium and high concentrations. This is sufficient to show that ISL and LCB ameliorate BDE-47-induced apoptosis in RAW264.7 cells.

Oxidative stress caused by ROS accumulation is one of the main factors in apoptosis. The exposure of the body to organic pollutants can cause mitochondrial damage and oxidative stress, leading to cell dysfunction and apoptosis [47]. Oxidative stress means that the REDOX balance of the body is disrupted, and a large number of ROS are produced [48]. Excessive reactive oxygen species can cause damage to the body. ROS and nitric oxide (NO) produced through cellular oxidative stress interact with surrounding molecules and affect the Redox balance in the body, which causes protein, DNA, and membrane damage, eventually leading to cell stress and death [49]. However, ROS in moderation also benefit immune cells and are involved in cell function and signaling [50]. This study found that exposure to polychlorinated biphenyls significantly increased ROS levels in both female and male zebrafish [51]. Studies have found that ISL and LCB have antioxidant effects. ISL attenuates the massive production of ROS in rat liver cells (HSC-T6 cells) [52], microglial cells (BV-2 cells) [53], and mouse hippocampal neuronal cells (HT 22 cells) [54]. LCB could reduce apoptosis by reducing ROS accumulation in PC-12 cells, maintaining nervous system health [55]. In addition, LCB protects against ethanol-induced oxidative stress in hepatocytes by influencing the activation of Erk signaling and promoting the nuclear transfer of Nrf2 [56]. In a CCL_4_-induced mouse hepatotoxicity model, LCB promoted the secretion of SOD, MDA, and GSH, alleviating liver damage [57]. We detected the levels of ROS in the cells, and the flow cytometry findings show that, after 24 h of BDE-47 acting on the cells, the levels of ROS increased. BDE-47 also decreased the activity levels of catalase and superoxide dismutase and the content of reduced glutathione in RAW264.7 cells. These results demonstrate that BDE-47 could cause an imbalanced redox state in cells. The above results are consistent with those of other studies [8,11,32,41]. Notably, compared with the ROS production in the BDE-47 group, the combination of low, medium, and high concentrations of ISL and LCB with BDE-47 could reduce ROS production to varying degrees, while increasing the expressions of antioxidant enzymes and GSH, alleviating cellular oxidative damage. 

The body protects itself by activating the endogenous antioxidant system to clear excess ROS [58]. Nrf2 is an endogenous antioxidant stress regulator; it binds to Keap1 in the cytoplasm under physiological conditions and is continuously degraded through ubiquitination. Keap1 binds to Nrf2, which prevents Nrf2 from entering the nucleus. Therefore, Keap1 is known as an inhibitor of Nrf2. When the body is stimulated by oxidative stress, the conformation of Keap1 changes, causing Nrf2 to become dissociated from it and activated. The activated Nrf2 enters the nucleus and binds to antioxidant reaction elements, thereby up-regulating the expressions of downstream antioxidant target genes (HO-1 and NQO1) to reduce oxidative stress and damage to the body [59]. Studies have shown that Licochalcone A activates the Nrf2/HO-1/NF-κB axis, inhibits pyrodeath and inflammatomes, and relieves osteoarthritis [60]. ISL activates the Nrf2 pathway through both classical and unclassical pathways, enhances antioxidant activity, and protects against liver damage [61]. ISL alleviates liver toxicity caused by rhubarb by activating the Nrf2 pathway and enhancing phase II metabolic enzymes [62]. In summary, activating the Nrf2 pathway can alleviate oxidative stress damage and thus protect the body. In this study, BDE-47 reduced the expressions of Nrf2 and its downstream antioxidant proteins (HO-1 and NQO1) in RAW264.7 cells, as confirmed in other studies [63,64,65]. ISL and LCB can reverse these effects. This indicates that treatment with ISL and LCB can activate the Nrf2 pathway, increasing the expressions of downstream antioxidant genes and proteins, thereby combating the oxidative stress damage and cell apoptosis caused by BDE-47. 

Organic pollutants cause inflammation in different body tissues or exacerbate the initiation of diseases [66,67]. The excessive secretion of proinflammatory factors can promote immune function disorders and apoptosis, and there is a close link with excessive inflammation [68]. The NF-κB pathway is a crucial element of the inflammatory response and controls both innate and adaptive immunity [69]. Nuclear factor κB regulates a variety of genes involved in inflammation. The formation of a complex between the NF-κB-inhibitory protein IκB-Alpha and NF-κB prevents the NF-κB from disintegrating. IKBKB is a kinase that catalyzes the degradation of IκB-Alpha, which allows NF-κB to enter the nucleus and exert proinflammatory actions [70]. It was found that BDE-47 up-regulated NF-κB in nucleus-induced renal inflammatory damage in mice [71]. The inhibition of NF-κB regulated the expressions of the IL-6 and IL-10 inflammatory factors and alleviated BDE-47-induced apoptosis and inflammatory damage in fish kidney cells [64]. In other studies, it was found that ISL could, through its anti-inflammatory effects, relieve the inflammation caused by renal fibrosis [72], pulmonary hypertension [73], and all diabetes diseases [74]. In this study, the BDE-47 stimulation of RAW264.7 cells could increase the expressions of the IKBKB and p-NF-κB/NF-κB proteins, while decreasing the expression of the IκB-Alpha protein, indicating that BDE-47 activates the NF-κB signaling pathway and induces inflammation in macrophages. Prior investigations have also clarified that BDE-47 can promote the expression of the NF-κB protein [16,63,71]. In our research, we discovered that ISL and LCB in conjunction with BDE-47 may significantly lower IKBKB protein expression while significantly raising the IκB-Alpha protein expression (*p* < 0.01). In addition, we also observed that this combination has a significant inhibitory effect on the expression of the p-NF-κB/NF-κB protein. These results suggest that the combination of ISL, LCB, and BDE-47 may have an important effect on the inflammatory response. Above all, it is concluded that different ISL and LCB treatments can inhibit the NF-κB pathway, reduce the release of inflammatory factors, and thus reduce cell apoptosis.

**Figure 7:**
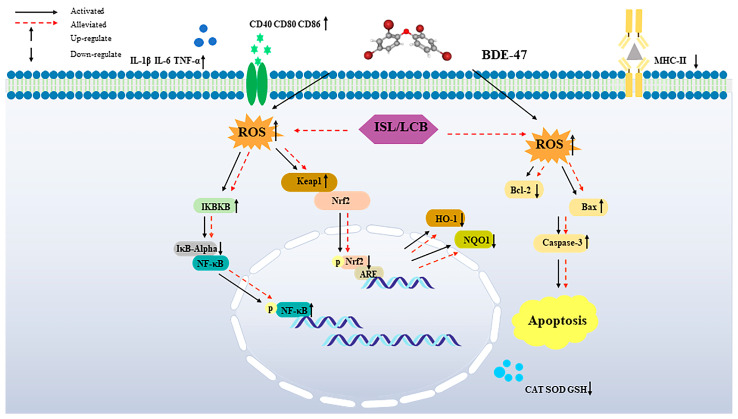
ISL and LCB alleviated BDE-47’s immunotoxicity in RAW264.7 cells.

This study confirmed that ISL and LCB increase the proliferation of BDE-47-exposed cells, thus alleviating the toxicity of BDE-47 in RAW264.7 cells. ISL and LCB alleviate the impairment of cellular immune function caused by BDE-47 and the apoptosis of BDE-47-infected cells. In addition, ISL and LCB activate the Nrf2 pathway, promoting the expression of HO-1 and NQO1 downstream. Therefore, the secretion of antioxidant substances (CAT, SOD, and GSH) is promoted, and the overproduction of ROS in BDE-47-infected cells is reduced, thus alleviating the oxidative damage of RAW264.7 cells induced by BDE-47. ISL and LCB inhibit the NF-κB pathway, thereby reducing the release of inflammatory factors in BDE-47-infected cells. The immune damage of RAW264.7 cells induced by BDE-47 is alleviated (Figure 7). According to our findings, ISL and LCB have detoxification effects on BDE-47-infected mouse macrophages. However, further studies are needed to reveal the effects of ISL and LCB on BDE-47-exposed organisms, which will help to elucidate the detoxification effects of ISL and LCB in many aspects.

## 5. Conclusions

This study confirmed that ISL and LCB can activate the Nrf2 pathway and inhibit the NF-κB pathway, relieving the oxidative stress and inflammatory responses caused by BDE-47, thereby alleviating immune injury. In addition, we demonstrated for the first time that LCB could mitigate the immunotoxicity induced by BDE-47. This study provided evidence that ISL and LCB alleviate the BDE-47-induced apoptosis of RAW264.7 cells, which not only elucidates the toxicological mechanism of BDE-47, but also demonstrates that the main component of licorice is a potential compound for treating BDE-47 poisoning. This study provides new clues for exploring this licorice component’s material basis and mechanism of action against the immunotoxicity of PBDEs and lays the foundation for mining the medicinal value and health care efficacy of licorice.

## Figures and Tables

**Table 1:** Sequences of experimental primers.

Target Gene	Accession Number	Forward (5′–3′)	Reverse (5′–3′)
*CD40*	NM_011611	ACCAGCAAGGATTGCGAGGCAT	GGATGACAGACGGTATCAGTGG
*CD80*	NM_009855	CCTCAAGTTTCCATGTCCAAGGC	GAGGAGAGTTGTAACGGCAAGG
*CD86*	NM_019388	ACGTATTGGAAGGAGATTACAGCT	TCTGTCAGCGTTACTATCCCGC
*MHC-II*	NM_207105	GTGTGCAGACACAACTACGAGG	CTGTCACTGAGCAGACCAGAGT
*CAT*	NM_009804	GCTCTCACATGGCTGCGAAGG	TCCTCAGGCTCGGCTTCACG
*SOD*	NM_011434	AACCAGTTGTGTTGTCAGGAC	CCACCATGTTTCTTAGAGTGAGG
*GSR*	NM_010344	CGGCGTGGAGGTGTTGAAGTTC	TGGTCGTGGTGGGCTTCCTAC
*Nrf2*	NM_010902	AAGCACAGCCAGCACATTCTCC	TGACCAGGACTCACGGGAACTTC
*Keap1*	NM_001110305	ATCCAGAGAGGAATGAGTGGCG	TCAACTGGTCCTGCCCATCGTA
*NQO1*	NM_008706	GCGAGAAGAGCCCTGATTGTACTG	AGCCTCTACAGCAGCCTCCTTC
*HO-1*	NM_010442	ACCGCCTTCCTGCTCAACATTG	CTCTGACGAAGTGACGCCATCTG
*IKBKB*	NM_010546	GCAGACTGACATTGTGGACCTG	ATCTCCTGGCTGTCACCTTCTG
*IκB-Alpha*	NM_010907	GCCAGGAATTGCTGAGGCACTT	GTCTGCGTCAAGACTGCTACAC
*NF-κB p65*	NM_009045	TCCTGTTCGAGTCTCCATGCAG	GGTCTCATAGGTCCTTTTGCGC
*GAPDH*	NM_008084	CATCACTGCCACCCAGAAGACTG	ATGCCAGTGAGCTTCCCGTTCAG

## Data Availability

The data are available on request due to privacy. The data presented in this study are available on request from the corresponding author.
